# Vancomycin Resistance in Enterococcus faecium from the Dallas, Texas, Area Is Conferred Predominantly on pRUM-Like Plasmids

**DOI:** 10.1128/msphere.00024-23

**Published:** 2023-03-20

**Authors:** Moutusee Islam, Belle Sharon, Ada Abaragu, Harita Sistu, Ronda L. Akins, Kelli Palmer

**Affiliations:** a Department of Biological Sciences, University of Texas at Dallas, Richardson, Texas, USA; b Methodist Health System, Dallas, Texas, USA; CDC

**Keywords:** *Enterococcus*, mobile genetic element, plasmid, vancomycin resistance, mobile genetic elements, plasmids

## Abstract

Vancomycin-resistant E. faecium (VREfm) is a significant public health concern because of limited treatment options. Genomic surveillance can be used to monitor VREfm transmission and evolution. Genomic analysis of VREfm has not been reported for the Dallas/Fort Worth/Arlington, TX, area, which is currently the 4th largest metropolitan area in the United States. Our study aimed to address this gap in knowledge by analyzing the genomes of 46 VREfm strains and 1 vancomycin-sensitive comparator collected during routine fecal surveillance of high-risk patients upon admission to a Dallas, TX, hospital system (August to October 2015). Thirty-one complete and 16 draft genome sequences were generated. The closed VREfm genomes possessed up to 12 extrachromosomal elements each. Overall, 251 closed putative plasmid sequences assigned to previously described and newly defined *rep* family types were obtained. Phylogenetic analysis identified 10 different sequence types (STs) among the isolates, with the most prevalent being ST17 and ST18. Strikingly, all but three of the VREfm isolates encoded *vanA*-type vancomycin resistance within Tn*1546*-like elements on a pRUM-like (*rep17*) plasmid backbone. Relative to a previously reported typing scheme for the *vanA*-carrying Tn*1546*, new variants of the Tn*1546* were identified that harbored a combination of 7 insertion sequences (IS), including 3 novel IS elements reported here (IS*Efa16*, IS*Efa17*, and IS*Efa18*). We conclude that pRUM-like plasmids are important vectors for vancomycin resistance in the Dallas, TX, area and should be a focus of plasmid surveillance efforts.

**IMPORTANCE** Vancomycin is an antibiotic used to treat infections caused by multidrug-resistant Gram-positive bacteria. Vancomycin resistance is common in clinical isolates of the Gram-positive pathogen Enterococcus faecium. Among E. faecium strains, vancomycin resistance genes can be disseminated by plasmids with different host ranges and transfer efficiencies. Surveillance of resistance plasmids is critical to understanding antibiotic resistance transmission. This study analyzed the genome sequences of VREfm isolates collected from the Dallas, TX, area, with particular focus on the mobile elements associated with vancomycin resistance genes. We found that a single plasmid family, the pRUM-like family, was associated with vancomycin resistance in the majority of isolates sampled. Our work suggests that the pRUM-like plasmids should continue to be studied to understand their mechanisms of maintenance, transmission, and evolution in VREfm.

## INTRODUCTION

Originally described as commensals of the healthy human gut, the enterococci attracted attention in the 1990s due to their alarming resistance to multiple antibiotics (1). Vancomycin-resistant enterococci (VRE) were first reported in 1988 in Europe (2) and in the United States in 1989 (3) and are a major concern (4). Vancomycin-resistant Enterococcus faecium (VREfm) is particularly concerning due to limited treatment options and widespread occurrence of vancomycin resistance among infection isolates of E. faecium (4).

Phylogenetic analysis of E. faecium strains revealed the presence of two major clades, referred to as clades A and B, with some evidence for subclades A1 and A2 within clade A (5, 6). Clade B E. faecium strains are mostly commensals of the healthy human gut. The multidrug-resistant E. faecium strains responsible for hospital outbreaks typically belong to clade A1, whereas animal gut commensals typically belong to clade A2 (6). Multilocus sequence typing (MLST) predates whole-genome-based phylogenetic analyses and has been used globally to classify E. faecium isolates by nucleotide sequence variations occurring in 7 housekeeping genes (7). Isolates are assigned to different sequence types (STs) based on the allelic variants. Most reported hospital outbreaks of VREfm have emerged from a single genetic lineage, referred to as clonal complex 17 (CC17), that was founded by ST17 (8).

Vancomycin resistance genes are carried within mobile elements in VREfm and can be horizontally disseminated (9–11). VanA-type resistance commonly occurs among VREfm strains, and VanA-type resistance genes are typically carried within Tn*1546* (12). Tn*1546* may be chromosomally integrated or plasmid borne. Different plasmid backbones carrying Tn*1546*, including Inc18, pRUM, and pLG1, have been reported worldwide (for examples, see references 10 and 13). Structural variations of Tn*1546* due to the presence of insertion sequences (IS), including IS*1251*, IS*1216*, IS*1485*, and IS*Ef1*, have also been reported. These previous studies characterized Tn*1546* structural variation by PCR mapping and then sequencing of the overlapping fragments (10, 13, 14). However, in the absence of a completely closed genome assembly, conclusive links between *vanA*, Tn*1546* variants, and specific plasmid backbones in VREfm can be difficult to achieve.

Surveillance of antibiotic resistance elements is important because changes in their host range and transfer frequency could evolve, which could impact clinical care. Utilization of whole-genome sequencing for epidemiological study provides an opportunity for multilevel analysis that includes detection of novel mobile genetic elements and monitoring DNA sequence changes in resistance gene vectors. The advent of long-read next-generation sequencing (NGS) by Pacific Biosciences and Oxford Nanopore Technologies (ONT) has made it possible to generate genome assemblies of a large number of bacterial isolates in a cost-effective manner. The combination of long (ONT in this study) and short (typically Illumina) reads generates high-quality, completely closed genome assemblies with fully assembled mobile genetic elements (15).

In this study, we analyzed phylogenetic relationships and plasmid diversity among a previously reported collection of VREfm isolates from the Dallas, TX, area (16). These isolates were recovered from rectal surveillance of high-risk patients upon admission to a Dallas hospital system. These represent colonization reservoirs from which future hospital outbreaks could emerge. Some results of our study were expected, including the predominance of CC17/clade A1 among the VREfm isolates. Other results highlight important areas for future study. Specifically, we note the high number of extrachromosomal elements in VREfm, with some isolates possessing 12 unique elements independent from the chromosome. How these multiple elements are coordinated and maintained in VREfm, and what they contribute to VREfm physiology, are largely unknown and are important topics for future investigation. Moreover, pRUM-like plasmids are the dominant carriers of vancomycin resistance genes among these VREfm isolates. This plasmid family should continue to be investigated in laboratory experiments to understand its maintenance and transmission mechanisms.

## RESULTS

### ST17 and ST18 VREfm isolates were prevalent in the collection.

Genome sequencing and hybrid assembly were performed for 47 E. faecium isolates with Oxford Nanopore MinION and Illumina technologies. Thirty-one closed and 16 draft genome assemblies were generated (see Data Set S1A in the supplemental material). In our previous analysis of these isolates using PCR, all isolates were found to be *vanA* positive and *vanB* negative, with the exception of the isolate 163-1, which had neither *vanA* or *vanB* and had a vancomycin MIC of 2 μg/mL by broth microdilution (16). We included 163-1 in this study for comparative purposes to evaluate its relationship to the *vanA*-positive isolates in the collection. After recovery from Spectra VRE rectal surveillance cultures, all 46 *vanA*-positive isolates sequenced in this study grew on agar supplemented with 256 μg/mL of vancomycin. Subsequent broth microdilution assays performed over a year later after genome sequencing and freezer restocking reconfirmed vancomycin resistance for all isolates except for 9-2 and 154-1, which had vancomycin MICs of ≤4 μg/mL. Likely, loss of vancomycin resistance elements during laboratory culture led to these phenotypes; however, this was not investigated further in this study.

10.1128/msphere.00024-23.1 DATA SET S1 Supplemental data files S1A to S1N. Download Data Set S1, XLSX file, 0.2 MB. Copyright © 2023 Islam et al. 2023 Islam et al. https://creativecommons.org/licenses/by/4.0/ This content is distributed under the terms of the Creative Commons Attribution 4.0 International license.

**FIG 1:**
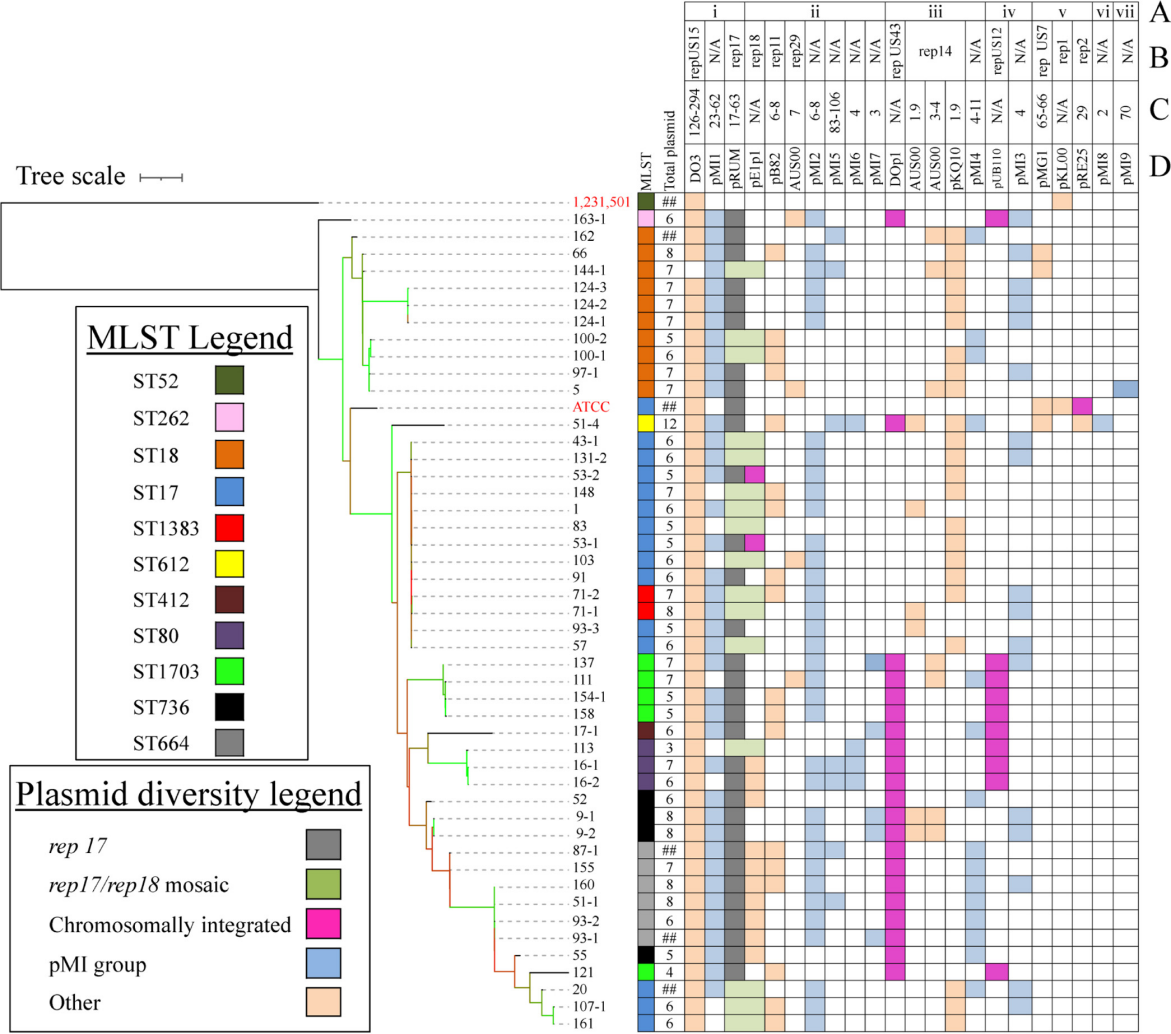
Phylogeny and plasmid content of Dallas E. faecium isolates. The phylogenetic tree of 49 E. faecium strains is based on an alignment of SNPs in 1,706 core genes. The branches colored green, gold, and red are supported by bootstrap values of >70, 40 to 60, and <30, respectively. The reference genomes E. faecium 1,231,501 and ATCC 700221 are labeled in red. Data for MLST, number of presumptive plasmids (shown as “##” for incomplete assemblies), and plasmid *rep* types are shown. Plasmid replicon conserved domains RepA_N (i), Rep_3 (ii), Rep_trans (iii), Rep1 (iv), Inc18 (v), Rep_2 (vi), and Rol_Rep_N (vii) (A), *rep* family (B), plasmid size ranges (C), and reference plasmid names (D) present in each strain are shown. A dark purple box indicates a chromosomally integrated plasmid whose size range was not determined. The *rep17* and mosaic of *rep17/rep18* are indicated with the gray and light green boxes, respectively. Plasmid groups defined in our study (pMI1 to pMI9; see main text) are highlighted in light blue. N/A, not applicable.

Phylogenetic analysis based on alignment of 1,706 core genes allowed us to assess the diversity of the VREfm isolates (Fig. 1). Two reference genomes, E. faecium 1,231,501 (5) and E. faecium ATCC 700221 (17), were included as representatives of E. faecium clades A2 and A1, respectively. E. faecium 1,231,501 is a human bloodstream isolate from the United States and is susceptible to vancomycin. E. faecium ATCC 700221 is a U.S. isolate and VanA-type VREfm control strain used in antimicrobial susceptibility testing. As expected, the Dallas VREfm isolates clustered with the clade A1 reference, E. faecium ATCC 700221.

**FIG 2:**
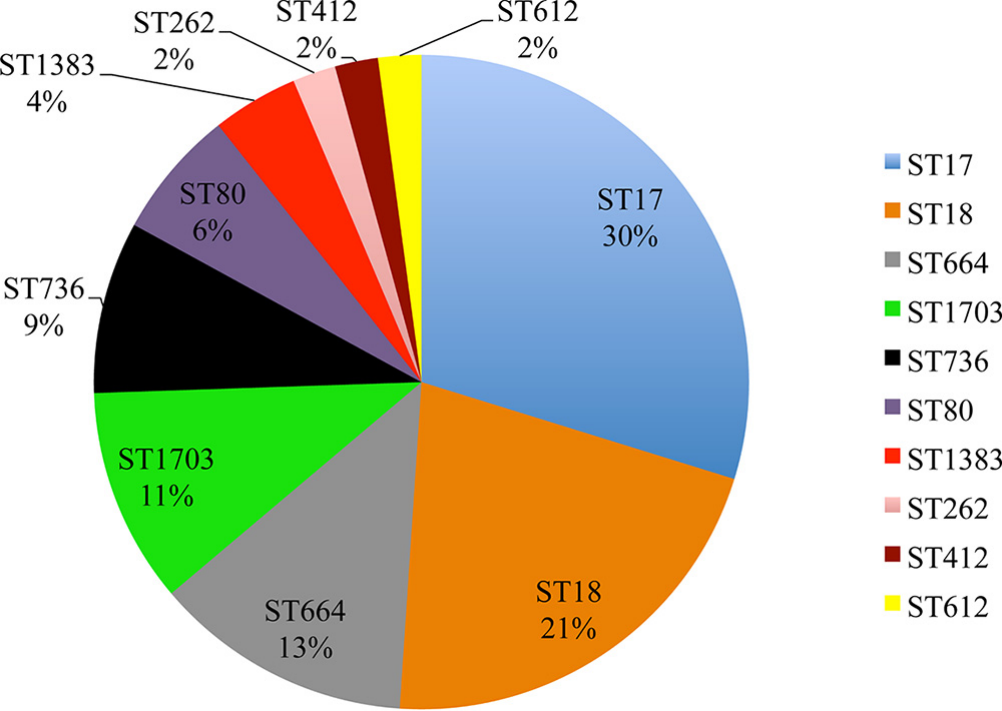
Sequence type (ST) distribution of Dallas E. faecium isolates. A total of 10 STs were observed across 47 isolates. The frequency of STs (percentage of total isolates) is shown.

Ten different sequence types (STs), all belonging to clonal complex 17 (CC17), were identified in the collection (Fig. 1 and 2). Isolates belonging to ST17 and ST18 were the most prevalent in the collection, comprising 50% of the isolates, which clustered together in the core genome tree (Fig. 1 and 2). Six STs (ST18, ST664, ST1703, ST736, ST80, and ST612) are double-locus variants of ST17. ST262 and ST412 are triple-locus variants and ST1383 is a single-locus variant of ST17 (Data Set S1D). ST1703, a double-locus variant of ST17 (*pstS* allele 20; novel *gyd* allele 70), is a novel ST in the MLST database and comprised 11% of the collection. We conclude that E. faecium isolates of multiple different STs, all within CC17, colonized the Dallas patients sampled at the time of the study.

We performed an additional analysis to determine how closely related the E. faecium strains in our collection are. All-versus-all average nucleotide identity (ANI) was calculated with all E. faecium assemblies as input (Data Set S1E). Dallas E. faecium isolates possess ≥98.98% ANI, consistent with the core gene phylogeny in Fig. 1. In most cases, strains isolated from the same patient were identical or nearly so. For example, the three isolates from patient 124 shared 99.99% ANI. Similar observations were made for isolates from patients 100, 16, 53, 71, and 9. Exceptions were patients 51 and 93, who each were colonized with different E. faecium STs (Fig. 1), also reflected in the ANI analysis of the isolates (≤99.4% ANI).

### Large numbers of plasmids were present in the Dallas isolates.

The plasmid content of the Dallas isolates was analyzed by an *in silico* detection method utilizing the conserved nucleotide sequences of replication initiation (*rep*) genes (18). Each contig of <2 Mbp in size in a closed genome assembly was considered a putative plasmid. Of the 259 closed circular putative plasmids, 141 were assigned to 10 known *rep* families described in the PlasmidFinder database. The remaining 118 putative plasmids were categorized into 9 novel plasmid groups (pMI1 to pMI9) and 8 other elements (discussed in the next section).

The Dallas isolates harbored variable numbers of putative plasmids (*n* = 3 to 12) of sizes ranging from 1.9 to 294 kb (Fig. 1; Data Set S1F). The most common elements were a megaplasmid (126 to 294 kb) and a pRUM-like plasmid (17 to 63 kb), which were each present in all isolates except 144-1, which lacked the megaplasmid. Both the reference strains (E. faecium 1,231,501 and ATCC 700221) harbored the megaplasmid, but 1,231,501 lacked the pRUM-like plasmid. Each of the megaplasmids harbored the *rep* gene *repUS15*, encoding a RepA_N protein motif. The pRUM-like plasmid harbored *rep17* or both *rep17* and *rep18*, which we refer to as a mosaic plasmid. An analysis of plasmid replicon enrichment among specific STs is presented in Text S1 and Data Set S1G.

10.1128/msphere.00024-23.2 TEXT S1 Supplemental methods and results. Download Text S1, PDF file, 0.1 MB. Copyright © 2023 Islam et al. 2023 Islam et al. https://creativecommons.org/licenses/by/4.0/ This content is distributed under the terms of the Creative Commons Attribution 4.0 International license.

### Uncategorized plasmids were sorted into 9 plasmid groups, pMI1 to pMI9.

PlasmidFinder (18) did not identify *rep* genes in a total of 118 circular entities. Of these 118 circular entities, 110 were established to be plasmids based on the presence of *rep* genes not currently represented in the PlasmidFinder database (see Materials and Methods). The remaining 8 circular entities were designated putative excision products of genomic islands (Text S1; Data Set S1H and I), including a 5-kb circular fragment of Tn*1546* in isolate 111.

The 110 plasmids not classifiable by PlasmidFinder were sorted into 9 plasmid groups, pMI1 to pMI9, based on phylogenetic analyses of the *rep* genes (Fig. S1; Data Set S1J). The nucleotide sequences of the *rep* genes and their corresponding amino acid sequences shared >95% identity within each pMIX plasmid group, except for pMI4 (Table 1). The clustering of the *rep* gene sequences belonging to the pMI4 plasmid group supported their inclusion in a single group, despite lower pairwise sequence identities (Fig. S1). A total of 6 Rep protein families (RepA_N, Rep_3, Rep_1, Rep_trans, Rol_rep_N, and Rep_2) were encoded by the *rep* genes of the 9 pMIX groups (Data Set S1J). Specifically, the *rep* genes of pMI1 and pMI3 encoded protein families RepA_N and Rep_1, respectively. The *rep* genes of pMI2, pMI5, pMI6, and pMI7 encoded the protein family Rep_3. The *rep* genes of pMI4 and pMI8 encoded protein families Rep_trans and Rep_2, respectively. The *rep* of pMI9 was the only exception, encoding two Rep protein families, Rep_trans and Rol_rep_N (Table 1).

**TABLE 1:** Features of the pMIX group plasmids

Plasmid group	No. of isolates with plasmid group^*a*^	% of isolates harboring the plasmid^*b*^	Plasmid size range (kb)	SD of plasmid size within group	*rep* gene information				
					*rep* gene size (bp)	% identity within group (*rep* DNA)^*c*^	% identity within group (*rep* aa)^*d*^	*rep-*encoded protein family^*e*^	Pfam accession no.
pMI1	35	74	23–62	11.2	1,086	99.7	98.9	repA_N	PF06970.11
pMI2	32	68	6–8	0.3	951	100	100	Rep_3	PF01051.21
pMI3	17	36	3.6–4.3	0.2	990	100	100	Rep_1	PF01446.17
pMI4	11	23	4–11	2.3	972	79.4	67.9	Rep_trans	PF02486.19
pMI5	5	11	83–106	8.7	804	95.40	95.9	Rep_3	PF01051.21
pMI6	4	9	4	0	924	100	100	Rep_3	PF01051.21
pMI7	4	9	3	0	720	100	100	Rep_3	PF01051.21
pMI8	1	2	2	NA	603	NA	NA	Rep_2	PF01719.17
pMI9	1	2	70	NA	1215	NA	NA	Rol_Rep_N	PF18106.1
								Rep_trans	PF02486.19

a Total number of isolates among the 47 Dallas isolates harboring the particular pMIX group plasmids.

b The percentage of isolates harboring the specific plasmid group.

c Percent identical sites calculated from the MUSCLE sequence alignment of DNA.

d Amino acid (aa) identity of the *rep* genes belonging to the same pMIX group.

e NA, not applicable.

10.1128/msphere.00024-23.3 FIG S1 Alignment of replicon sequences in 110 pMIX plasmids. Nucleotide (*rep*) and amino acid sequences (Rep) were aligned with MUSCLE, and neighbor joining trees are shown. A total of 9 plasmid groups (pMI1 to pMI9) were assigned based on the clustering of sequences within the trees. Download FIG S1, PDF file, 1.6 MB. Copyright © 2023 Islam et al. 2023 Islam et al. https://creativecommons.org/licenses/by/4.0/ This content is distributed under the terms of the Creative Commons Attribution 4.0 International license.

Each of the Dallas isolates harbored 1 to 5 of the pMIX plasmid groups, except isolate 83 (ST17), which had none of them (Fig. 1). Neither of the two reference strains harbored any of the pMIX plasmid groups. pMI1 was the most frequently occurring pMIX plasmid group, identified in 75% of the isolates (Table 1). pMI8 and pMI9 were the rarest ones, found only in isolates 51_1 (ST612) and 5 (ST18), respectively. The pMIX plasmid groups were not enriched in any specific ST. We observed that plasmids within a very narrow size range (standard deviation, 0.2 to 2.3) were grouped together by this *rep* typing scheme (with the exception of pMI1), although plasmid size was not taken into consideration in assigning plasmid groups.

We used PLSDB (19, 20) to compare representative *rep* sequences from the pMIX plasmid groups with existing plasmids in the database (as of January 2023). PLSDB compiles plasmid sequences and their associated metadata, including PlasmidFinder and other analyses. This was done as an additional check on our analysis, as ours was initially performed in 2018-2019. We reasoned that the pMIX typing scheme would be most useful if it encompassed other previously untypeable plasmids in the database. We applied strict thresholds of 90% identity and 90% query coverage to filter BLASTn hits to the database. The results support pMI1, pMI3, pMI5, pMI6, and pMI8 as newly defined *rep* types that include other previously untyped *Enterococcus* plasmids from around the world (Data Set S1K). The only hit to pMI9 *rep* was the Dallas isolate from which it was defined. In this PLSDB analysis, pMI2 was assigned to *rep11a*, and pMI4 was assigned to *rep14b*. The pMI7 analysis was more complicated, with one group of identical hits (100% identity and query coverage) for many plasmids with no known *rep* type assigned and another group of hits with 100% identity but lower (98%) coverage that were assigned to *rep17*.

### Resistance genes were carried in both chromosomes and plasmids.

**FIG 3:**
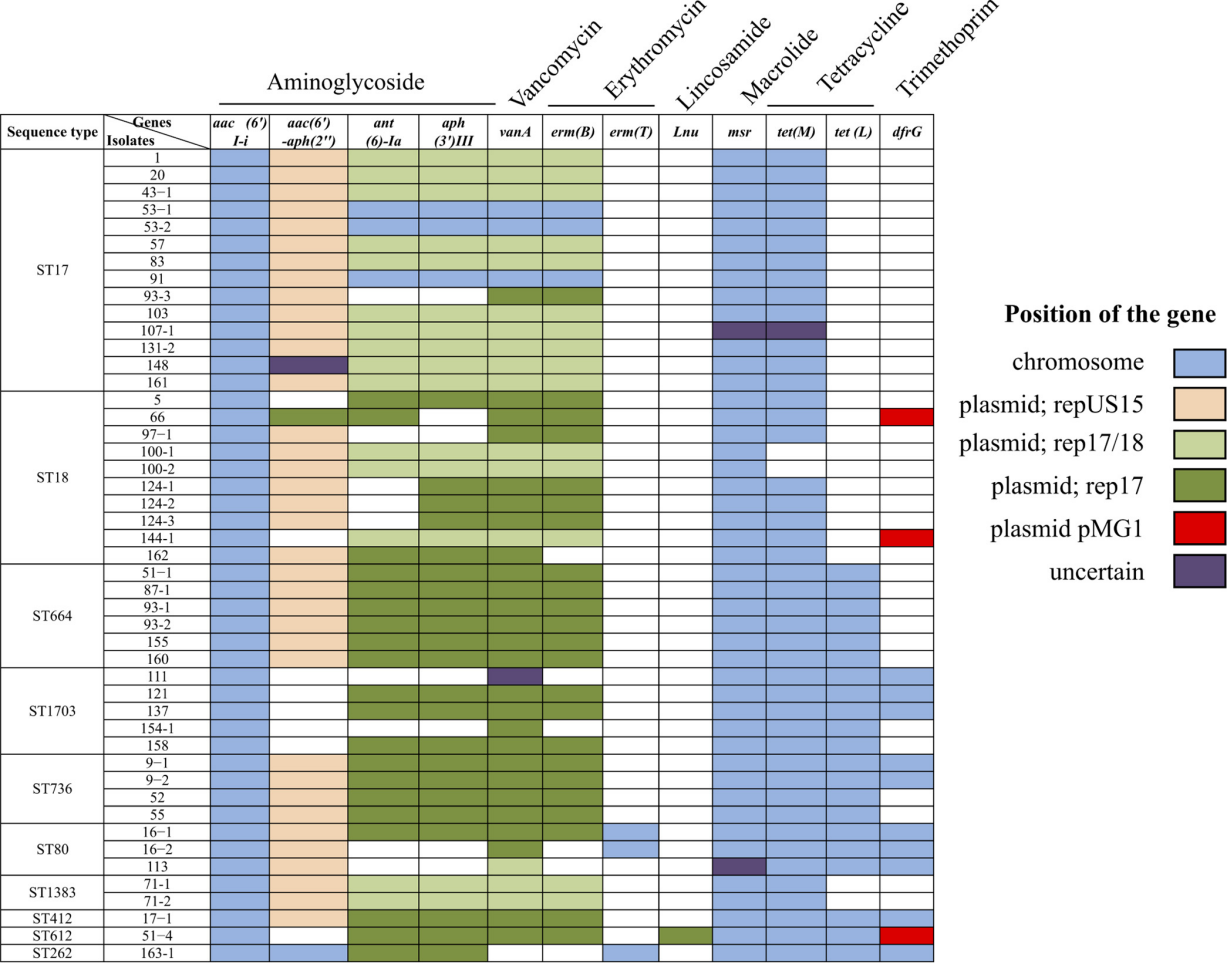
Distribution of predicted antibiotic resistance genes among Dallas isolate genomes. Shown are 12 resistance genes {6′-*N*-aminoglycoside acetyltransferase [*aac(6′)I-i*], bifunctional enzyme 6′-aminoglycoside acetyltransferase-2″-aminoglycoside phosphotransferase [*aac(6′)-aph(2″)*], aminoglycoside nucleotidyltransferase [*ant(6)-Ia*], *aph(3′)III*, *vanA*, *erm*(B), *erm*(T), *lnu*, acquired macrolide resistance-like protein [*msr*], *tet*(M), *tet*(L), and dihydrofolate reductase [*dfrG*]}, and the antibiotics they provide resistance to. A colored box indicates the presence of a certain resistance gene in the isolate. The boxes are color-coded to indicate the location of the gene (chromosome or plasmid).

Different combinations of 12 resistance genes predicted to provide resistance against 5 classes of antibiotics (aminoglycosides, glycopeptides, macrolides, tetracycline, and trimethoprim) were identified among the Dallas isolates (Fig. 3; Data Set S1L; Text S1). The most commonly occurring resistance genes were *aac(6′)I-1* (100% of isolates), *msr* (100%), *vanA* (95%), *tetM* (95%), *ermB* (85%), and *aph(3′)* (85%). These resistance genes were carried on the chromosome and primarily on only two plasmid types, the pRUM-like plasmid group and the megaplasmid. The pRUM-like plasmids predominantly carried 4 of the resistance genes [*ant(6)-Ia*, *aph(3′)III*, *vanA*, and *ermB*] conferring resistance against aminoglycosides, vancomycin, and erythromycin (Fig. 3). The *repUS7* plasmid (pMG1) was found among only 3 of the Dallas isolates—66 (ST18), 144-1 (ST18), and 51-4 (ST612)—and carried *dfrG*, for trimethoprim resistance. We note that antibiotic resistance of these isolates was not phenotypically assessed in this study, other than for vancomycin.

All of the Dallas isolates, except for 163-1, carried VanA-type vancomycin resistance genes. The *vanA* genes possessed 99.9% nucleotide and 99.7% amino acid sequence identity with the reference *vanA* sequence (GenBank accession number AAA65956.1 (Data Set S1L). The reference sequence is encoded within the transposon Tn*1546* (GenBank accession number M97297), originally described for the human fecal isolate E. faecium BM4147, collected from France in 1988 (21) (Fig. S2). A classical structure of the *vanA* gene cluster, consisting of *vanHAX* and *vanYZ* carried within Tn*1546* elements, was observed among the Dallas VREfm isolates, except for isolate 17-1, which lacked the genes *vanY* and *vanZ*. The Tn*1546* structure varied among the isolates in terms of insertion site (chromosome versus plasmid), IS elements, and point mutations, discussed further below.

10.1128/msphere.00024-23.4 FIG S2 Tn*1546* structure and regulatory regions. The Tn*1546* structure consisting of IR_L_ (inverted repeat, left), ORF1 (transposase), ORF2 (resolvase), regulatory and resistance genes (*vanR*, *vanS*, *vanH*, *vanA*, *vanX*, *vanY*, and *vanZ*), and IR_R_ (inverted repeat, right) are delineated. The 5′ region of IR_L_ marks the starting position (1), while the 3′ region of the IR_R_ boundary marks the end position (10851) of Tn*1546.* The positions of the *vanR* promoter region at 3803 and 3999 (−173 and +24 regions), the VanR binding site at positions 5846 and 5879, and the 70-bp VanR-interacting region at positions 5935 and 6004 are indicated. The figure is not drawn to scale. Download FIG S2, PDF file, 0.04 MB. Copyright © 2023 Islam et al. 2023 Islam et al. https://creativecommons.org/licenses/by/4.0/ This content is distributed under the terms of the Creative Commons Attribution 4.0 International license.

### Tn*1546* was borne on pRUM-like plasmids in most isolates.

**FIG 4:**
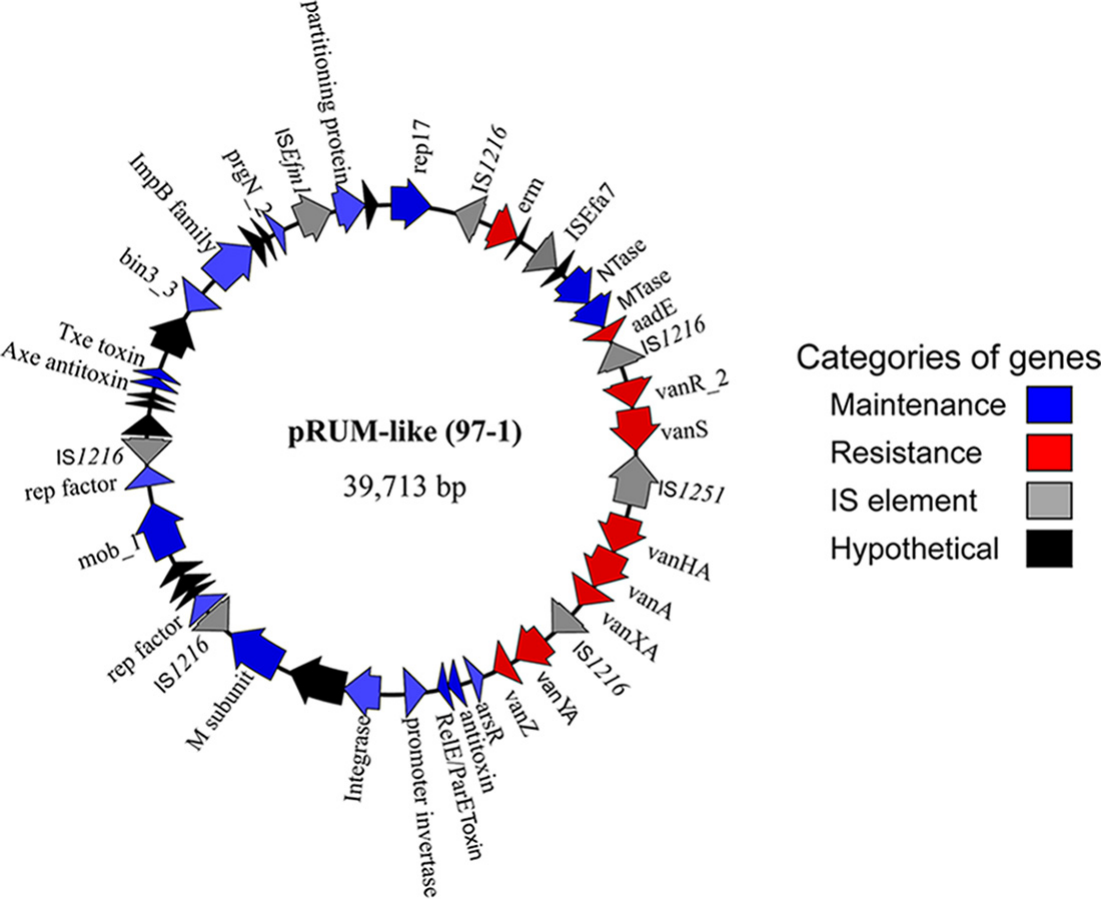
Genetic map of a representative group 2a pRUM-like plasmid. The 39-kb plasmid p97_1_39kb (GenBank accession number CP066571) from the Dallas VREfm isolate 97-1 is shown. Arrows represent genes. The genes are color-coded based on their predicted activity. Maintenance genes are predicted to be responsible for general maintenance of the plasmid within the bacterial host. Resistance genes provide antibiotic resistance.

The complete Tn*1546* was carried by the pRUM-like plasmid in 42 of 46 *vanA*-positive isolates. A representative pRUM-like plasmid from our collection is shown in Fig. 4. Isolate 111 did not carry the entire Tn*1546* in the pRUM-like plasmid; rather, it carried *vanRS* in a 17-kb pRUM-like plasmid, while *vanHAX* was carried within a separate 5-kb circular element (te111_5kb) (Data Set S1H). Tn*1546* was chromosomally borne in 3 isolates, 53-1, 53-2, and 91, each belonging to ST17, with all three strains also harboring a pRUM-like plasmid lacking Tn*1546* (Table 2).

**TABLE 2:** Features of the pRUM-like plasmid groups

Plasmid group	MLST	Isolate	Rep^*a*^		TA^*a*^		Size (bp)	Size (kbp) (avg ± SD)
			*rep17*	*rep18*	Axe-txe	relE		
Group 1	ST17	1					44,323	44 ± 1.9
		20					44,443	
		43-1					44,730	
		57					44,421	
		83					44,442	
		103					48,758	
		107-1					41,004	
		131-2					43,860	
		148					45,965	
		161					44,443	
	ST18	100-1					43,236	
		100-2					43,236	
		144-1					47,773	
	ST1383	71-1					44,420	
		71-2					44,420	
	ST80	113		a			41,153	
Group 2a	ST18	5					45,647	37 ± 4.3
		66					38,373	
		97-1					39,713	
		124-1					37,877	
		124-2					37,877	
		124-3					37,877	
		162					34,583	
	ST80	16-1					41,600	
		16-2					33,441	
	ST17	93-3					34,144	
	ST612	51-4					29,409	
Group 2b	ST664	51-1					61,762	59 ± 3
		87-1					57,116	
		93-1					63,108	
		93-2					63,108	
		155					57,134	
		160					57,135	
Group 3	ST736	9-1					38,857	38 ± 2.6
		9-2					38,857	
		52					36,284	
		55					36,284	
	ST1703	121					42,671	
Group 4	ST1703	111					17,982	28 ± 7.4
		137					35,487	
		154-1					22,729	
		158					30,941	
	ST412	17-1					33,158	

a The presence of the gene is indicated by shaded boxes. The pRUM-like plasmid from isolate 113 harbored *rep18a* (reference gene; GenBank accession number AB158402) and is symbolized as “a” in the corresponding box. The *rep18* gene for the rest of the resistance plasmids was *rep18b* (reference gene; GenBank accession number CP018068). TA, toxin-antitoxin.

**FIG 5:**
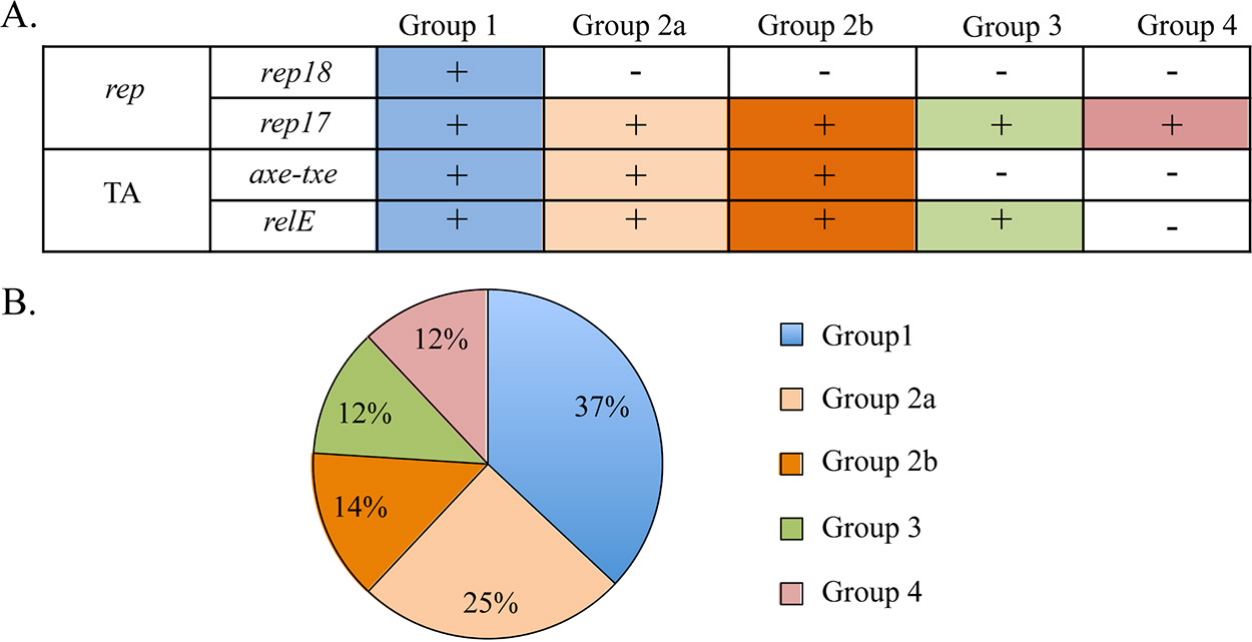
Classification and distribution of pRUM-like plasmid groups. (A) pRUM-like plasmids were classified based on the presence or absence (indicated by a plus or minus, respectively) of 2 replication modules (*rep17* and *rep18*) and 2 stability modules (toxin-antitoxin system; TA_axe-txe_ and TA_relE_). (B) Frequency of the pRUM-like plasmid groups among 43 Dallas isolates.

We categorized the 43 pRUM-like plasmids encoding Tn*1546* or a fragment of Tn*1546* (for isolate 111) into four groups based on the presence or absence of replication and stability modules on their backbones (Table 2; Fig. 5), per a previously described typing scheme (14). For the plasmids in our collection, two toxin-antitoxin (TA) systems were observed in different distributions, *axe-txe* (TA_axe-txe_) and a *relE-parE* family toxin associated with an uncharacterized antitoxin component (TA_relE_) (Table 2). Both of the TA systems were actively expressed in two representative VREfm isolates (Fig. S3).

10.1128/msphere.00024-23.5 FIG S3 Toxin-antitoxin (TA) systems are expressed during culture in laboratory medium. mRNA expression levels of antitoxin and toxin components of TA_axe-txe_ and TA_relE_ were quantified by RT-qPCR in the isolates VREfm1 and VREfm5. The *y* axis shows the expression levels relative to the housekeeping gene 16S rRNA. Download FIG S3, PDF file, 0.03 MB. Copyright © 2023 Islam et al. 2023 Islam et al. https://creativecommons.org/licenses/by/4.0/ This content is distributed under the terms of the Creative Commons Attribution 4.0 International license.

### Plasmid groups.

**(i) Group 1 plasmids (*n* = 16).** Group 1 plasmids possess *rep17*, *rep18*, and both TA_axe-txe_ and TA_relE_. All the group 1 plasmids carried *rep18b* (GenBank accession number CP018068), except isolate 113, which carried *rep18a* (GenBank accession number AB158402). The plasmids had a narrow size range (44 to 48 kb) and were present in VREfm isolates from ST17, ST18, ST1383, and ST80.

**(ii) Group 2 plasmids (*n* = 17).** Group 2 plasmids possess *rep17* and both TA_axe-txe_ and TA_relE_. The group 2 plasmids were classified further based on size and the genetic background of the isolates carrying them. Group 2a plasmids (29 to 45 kb) were carried by the reference strain ATCC 700221 and isolates belonging to ST18 (5, 66, 97-1, 124-1, 124-2, 124-3, and 162), ST80 (16-1 and 16-2), ST17 (93-2), and ST612 (51-4). The comparatively larger (57 to 63 kb) group 2b plasmids were present in all 6 isolates belonging to ST664 (51-1, 87-1, 93-1, 93-2, 155, and 160). Gene presence/absence analysis using Roary (22) identified a set of genes that are specific to group 2b plasmids (Data Set S1M), including an ~7.5-kb region encoding helix-turn-helix XRE family-like proteins (cd00093) inserted immediately upstream of the *van* gene cluster (Fig. S4).

10.1128/msphere.00024-23.6 FIG S4 Unique region in Group 2b pRUM-like plasmids. (A) Alignment of the pRUM-like plasmids from groups 2a and 2b. The alignment was generated by progressive Mauve algorithm from Geneious. The *van* gene cluster is highlighted with a red box. The region containing the helix-turn-helix XRE family-like DNA binding protein is indicated at the base of the alignment. This region is unique to the group 2b resistance plasmids. (B) Sketch of the region unique to group 2b is presented. Arrows indicate coding regions. Yellow arrows indicate predicted helix-turn-helix domain-containing proteins. Black arrows indicate hypothetical genes. IS elements are represented by purple arrows. Download FIG S4, PDF file, 0.2 MB. Copyright © 2023 Islam et al. 2023 Islam et al. https://creativecommons.org/licenses/by/4.0/ This content is distributed under the terms of the Creative Commons Attribution 4.0 International license.

**(iii) Group 3 plasmids (*n* = 5).** Group 3 plasmids possess *rep17* and TA_relE_ and have a size range of 36 to 42 kb. They were carried by ST736 isolates (isolates 9-1, 9-2, 52, and 55) and one ST1703 isolate (isolate 121).

**(iv) Group 4 plasmids (*n* = 5).** Group 4 plasmids possess *rep17* and no known stability modules and have a size range of 17 to 35 kb. These plasmids were carried by isolates belonging to the novel genetic background ST1703 (111, 137, 154-1, and 158) and by a single ST412 isolate (17-1).

We further analyzed the pRUM-like plasmids in our collection using two approaches: a core genome phylogeny based on sequence variation in 7 pRUM-like plasmid core genes (*rep17*, IS*1216*, *vanS*, and genes for ImpB/MucB/SamB family protein, hypothetical protein, plasmid partitioning protein, and replication control protein PrgN) (Fig. S5) and an analysis of ANI (Fig. S6; Data Set S1N). The pRUM-like plasmids form two clusters in the phylogenetic tree (Fig. S5), which correspond to the presence/absence of TA*_axe-txe_*. The pairwise ANI of the pRUM-like plasmids ranged from 93.9 to 100%. ANI analysis identified well-defined clusters of plasmids with high percent ANI that correspond to plasmid groups 2b, 3, and 4, while groups 1 and 2a were intermixed (Fig. S6). This is also consistent with the presence/absence of TA*_axe-txe_* being an important discriminator for grouping of pRUM-like plasmids.

10.1128/msphere.00024-23.7 FIG S5 pRUM-like plasmid core genome phylogeny. The RAxML phylogeny is based on an alignment of SNPs in the 7 core genes found in 40 pRUM-like plasmids. The tips of the tree are labeled with E. faecium isolate names. The tree tip labels are color-coded according to the plasmid groups 1, 2a, 2b, 3, and 4. The branches colored green and red are supported by bootstrap values of >70, and <30, respectively. Download FIG S5, PDF file, 0.1 MB. Copyright © 2023 Islam et al. 2023 Islam et al. https://creativecommons.org/licenses/by/4.0/ This content is distributed under the terms of the Creative Commons Attribution 4.0 International license.

10.1128/msphere.00024-23.8 FIG S6 ANI analysis of pRUM-like plasmids. Percent ANI is color-coded as shown in the key. Plasmid groups 2b, 3, and 4 (with the exception of the pRUM-like plasmid from isolate 111, at top) are shown in boxes. Plasmids belonging to group 2a are indicated with red stars. All other plasmids belong to group 1. Download FIG S6, PDF file, 0.2 MB. Copyright © 2023 Islam et al. 2023 Islam et al. https://creativecommons.org/licenses/by/4.0/ This content is distributed under the terms of the Creative Commons Attribution 4.0 International license.

**FIG 6:**
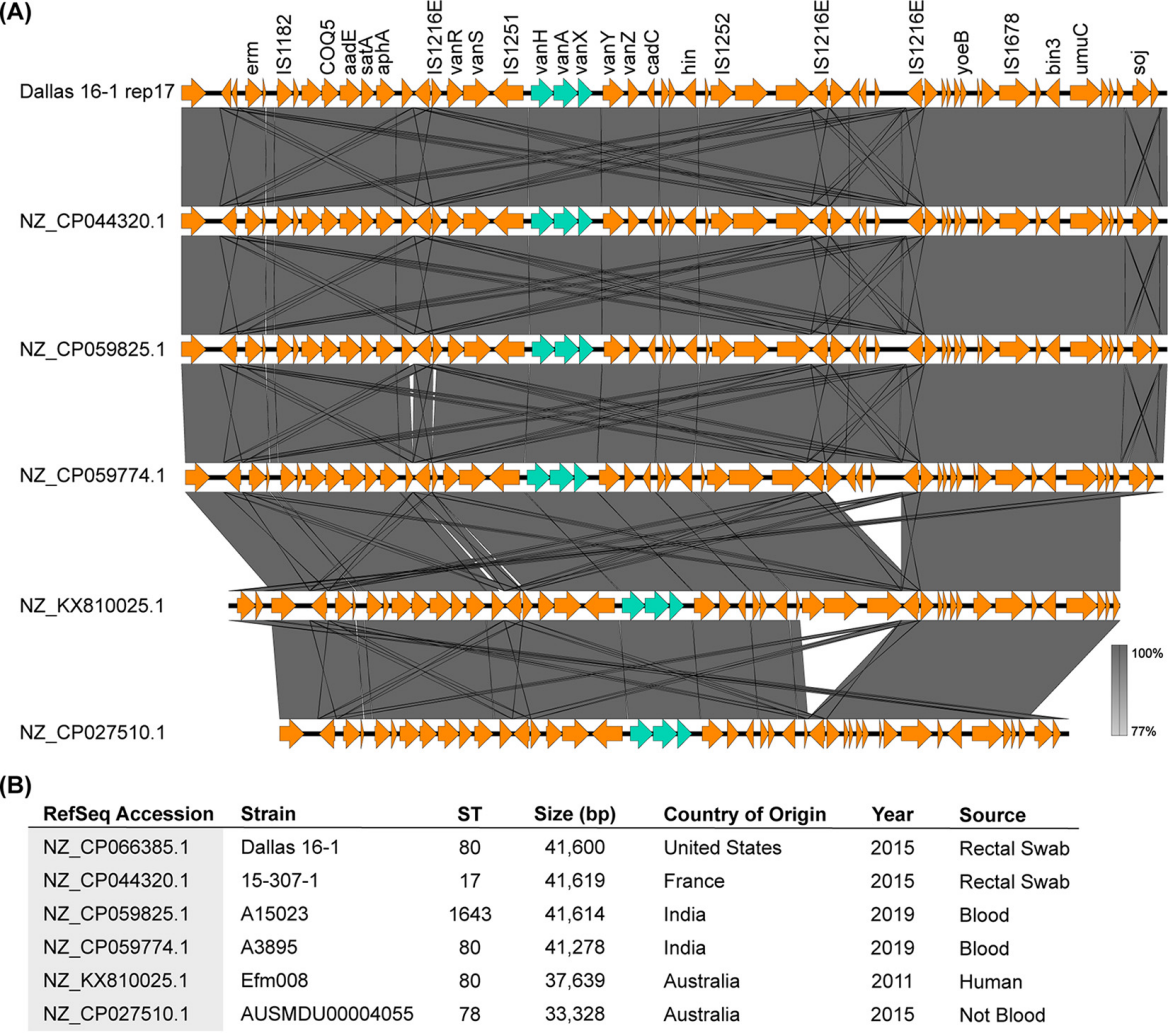
Cluster of pRUM-like plasmids identified from PLSDB with 99.9% ANI. (A) BLASTn alignment of plasmid sequences with Easyfig. (B) Data for E. faecium strains and plasmids, arranged in the same order as in the alignment.

Finally, we compared pRUM-like/*rep17* plasmids from the Dallas collection with other *rep17* plasmids that have previously been published. PLSDB was queried to obtain circular plasmids assigned to genus *Enterococcus* and to *rep17*. A strict cutoff of ≥99.9% pairwise ANI was applied, which identified a cluster of closely related pRUM-like/*rep17* plasmids from different sources (Fig. 6). These plasmids have ≥99.9% ANI, are from geographically distinct regions (United States [this study], France [23], India [24], and Australia [25, 26]), and are present in different E. faecium STs. The most closely related are the pRUM-like/*rep17* plasmid from Dallas isolate 16-1 and the *rep17* plasmid from the VREfm strain 15-307.1. These plasmids have identical gene contents and differ by single nucleotide polymorphisms (SNPs) and deletions/insertions at 10 sites. VREfm 15-307.1 was isolated in France in 2015 from a rectal swab of a hospitalized patient, which is similar to the collection of Dallas 16-1 in 2015.

### Dallas VREfm isolates harbored 17 variants of Tn*1546*.

**FIG 7:**
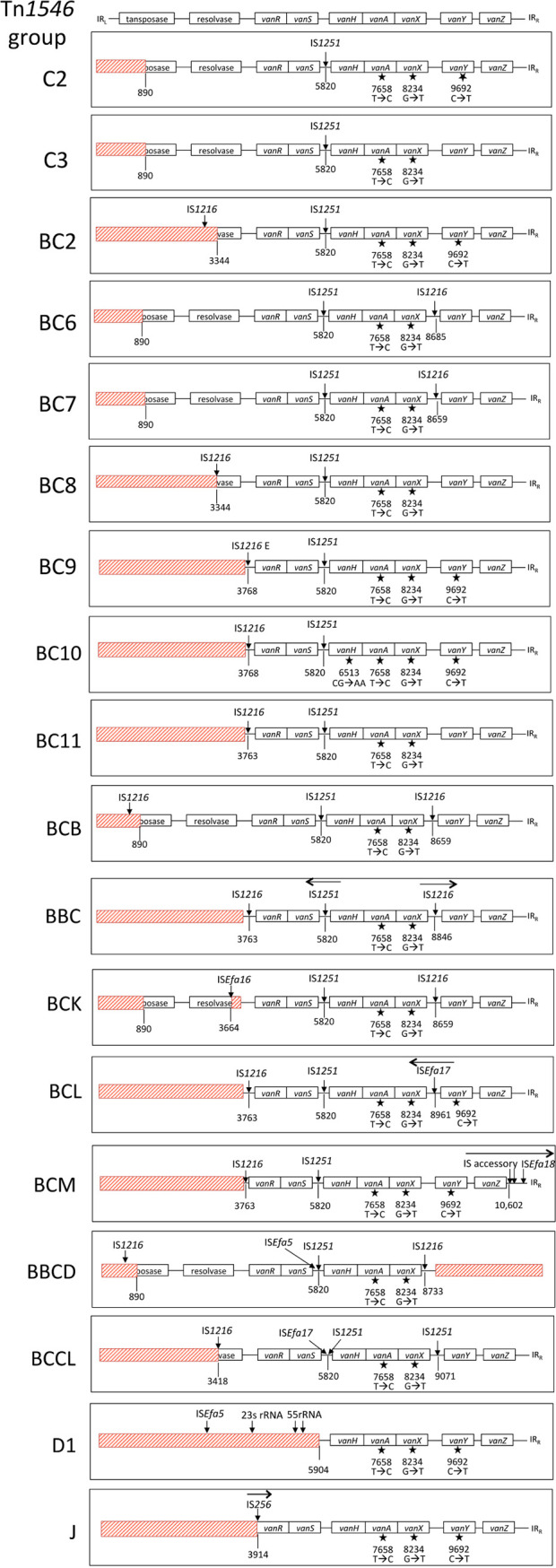
Tn*1546* structural variations in the Dallas VREfm isolates. A reference Tn*1546* structure that includes transposase, resolvase, and *van* gene cluster flanked by IR_L_ and IR_R_ (inverted repeats, left and right, respectively) is delineated at the top. The Tn*1546* groups are represented below the reference Tn*1546*, and the name of the group (C3, BC2, BC6, BC7, BC8, BC9, BC10, BC11, BCB, BBC, BCK, BCL, BCM, BBCD, BCCL, D1, or J) is shown on the left. Red boxes represent deleted regions relative to the reference Tn*1546*. Vertical lines with numbers at the bottom represent the nucleotide position within the reference Tn*1546.* IS elements are represented by downward arrows. Black stars indicate point mutations. The figure is not drawn to scale.

The Tn*1546* region of the Dallas isolates varied from the typical structure, which consists of ORF1 (transposase), ORF2 (resolvase), and the *van* genes flanked by IR_L_ (inverted repeat, left) and IR_R_ (inverted repeat, right) (Fig. 7). A total of 17 different sequence variations in the Tn*1546* were observed among 45 of the Dallas isolates (Fig. 7; Table 3). These 17 variations were classified using a nomenclature system developed previously (13) based on the presence of IS elements, point mutations, and deletions relative to the reference, Tn*1546*. VREfm isolates belonging to the same Tn*1546* group shared 99.8% to 100% nucleotide sequence identity in the Tn*1546* region. Isolate 111 was excluded from this grouping since it harbored a fragmented Tn*1546* occurring in a pRUM-like plasmid and another circular entity (Data Set S1H). The Tn*1546* variation was mostly associated with the distribution of 7 different IS elements (IS*1251*, IS*1216*, ISEfa*16*, IS*Efa5*, IS*Efa17*, IS*256*, and IS*Efa18*), three of which were novel to the Dallas isolates. Most of the Tn*1546* groups described here are novel relative to those previously reported in the literature, except for group BC2, which was reported previously in Poland (13). The most predominant Tn*1546* groups were BC9 (*n* = 14) and J (*n* = 6). The BC9 variety was observed in ST17 (*n* = 9) and ST18 (*n* = 5) isolates. The reference strain, ATCC 700221, harbored the C2 structural variant of Tn*1546* that was described previously (13) but was not present in the Dallas VREfm isolates. The presence of different pRUM groups and Tn*1546* types across the phylogeny of E. faecium isolates in this study is presented in Fig. S7.

**TABLE 3:** Features of the Tn*1546* groups observed among the Dallas isolates

pRUM-like plasmid group	Tn*1546* group	Isolate	% identity within Tn*1546* group^*a*^	No. of isolates in Tn*1546* group	IS element(s)^*b*^	Point mutation^*c*^				
						*vanH* (6513, 6514 CG → AA) Arg → Lys	*vanA* (7658 T → C) Val→ Ala	*vanX* (8234 G → T) Lys→ Asn	*vanY* (9692 C → T) Pro → Leu	*vanY* (9566 G → A) Gly → Glu
1	BCL	43-1	NA	1	IS1216, IS1251, ISEfa17					
	BC8	113	NA	1	IS1216, IS1251					
	BC11	71-1	99.80	3	IS1216, IS1251					
		71-2								
		144-1								
	BC9	1	99.80	14	IS1216, IS1251					
		20								
		57								
		83								
		100-1								
		100-2								
		103								
		107-1								
		131-2								
		148								
		161								
2a		5								
		66								
		162								
	BC2	16-1	100	3	IS1216, IS1251					
		16-2								
		51-4								
	BBC	97-1	NA	1	IS1216, IS1251					
	BCM	93-3	NA	1	IS1216, IS1251, ISEfa18					
	BCCL	124-1	100	3	IS1216, ISEfa17, IS1251, IS1251					
		124-2								
		124-3								
2b	J	51-1	100	6	IS256					
		87-1								
		93-1								
		93-2								
		155								
		160								
3	C3	52	100	2	IS1251					
		55								
	BC6	9_1	100	2	IS1251, IS1216					
		9_2								
	BC7	121	NA	1	IS1251, IS1216					
4	BCB	154-1	100	2	IS1216, IS1251, IS1216					
		158								
	BCK	137	NA	1	ISEfa16, IS1251, IS1216					
	BBCD	17-1	NA	1	IS1216, ISEfa5, IS1251, IS1216					
Chromosome-integrated Tn*1546*	D1	91-1	NA	1	ISEfa5					
	BC10	53-1	100	2	IS1216, IS1251					
		53-2								

a Nucleotide sequences of the Tn*1546* region for the Tn*1546* groups, including more than one isolate, were aligned and percent identity was determined.

b The IS elements present in the Tn*1546* group are listed.

c Point mutations in the *van* gene cluster relative to the reference Tn*1546* (GenBank accession number M97297). The presence of a point mutation is represented by a shaded box.

10.1128/msphere.00024-23.9 FIG S7 Core genome phylogeny, MLST, pRUM-like plasmid groups, and Tn*1546* types. The tree is as shown in Fig. 1 in the main text. The presence of pRUM-like plasmids of 5 different types (1, 2a, 2b, 3, and 4) is indicated by the symbol “❖.” Isolates 163-1 and the reference E. faecium 1,231,501 lack *vanA-*carrying Tn*1546*. Isolates 53-1, 53-2, and 91 have chromosomally integrated Tn*1546*. In all of these cases a variant of pRUM-like plasmid exists in the genome but they are not denoted as *van* plasmids, except for E. faecium 1,231,501, for which plasmid information is unavailable due to its incomplete genome assembly. Structural variants of Tn*1546* are indicated with the symbol “✚.” The symbol “✔” indicates chromosomally integrated Tn*1546*. The absence of Tn*1546* in the isolates 163-1 and 1,231,501 is indicated by “N/A.” The *vanA* operon was present in two different contigs in isolate 111; therefore, Tn*1546* was considered fragmented. Download FIG S7, PDF file, 0.1 MB. Copyright © 2023 Islam et al. 2023 Islam et al. https://creativecommons.org/licenses/by/4.0/ This content is distributed under the terms of the Creative Commons Attribution 4.0 International license.

Specific structural variations of the Dallas Tn*1546* elements are described further in Text S1.

### Potential VREfm patient-patient transmission.

We used our genome data to look for identical E. faecium clones colonizing different patients. This would be suggestive of prior transmission of VREfm between patients in our area. We found one possible instance supported by our genomic data. E. faecium isolates from patients 55 and 52 were of identical ST (Fig. 1), the same pRUM plasmid size and group (Table 2), and the same Tn*1546* group (and were the only members of the group in this study) (Table 3) and possess 99.99% ANI overall (Data Set S1E). Their pRUM plasmids possess >99.99% ANI (Data Set S1N), differing by one SNP. However, the total genome sizes for these two strains differ. The isolate from patient 55 has a closed genome that is 1,026 bp longer than the draft genome of the patient 52 isolate (Data Set S1A).

## DISCUSSION

This study provided a snapshot of fecal VREfm colonizing hospitalized patients in Dallas, TX, from August to October 2015. VREfm isolates from previously described and new STs were detected. The most prevalent VREfm isolates, comprising 50% of those analyzed, belonged to ST17 and ST18. These STs were previously reported to be responsible for outbreaks in countries including Portugal (14), Spain (27), Ireland (28), Denmark (29), Columbia (30), Brazil (31), Canada (32). The other 8 STs identified among our collection (ST1383, ST612, ST412, ST80, ST1703, ST664, ST736, and ST262), were single, double, or triple locus variants of ST17. ST1703 is a novel sequence type described in this study. ST182, which caused an outbreak in San Antonio, TX, in the 1990s (33), was not detected in our collection.

All the plasmid-borne Tn*1546* elements in Dallas isolates were carried within pRUM-like plasmids that were clustered into 4 subgroups (1, 2a, 2b, 3, and 4), defined by the presence of the replication module *rep17* and a combination of other replication and stability modules. The patients from whom the isolates in this study were collected resided in different areas of Dallas and in unconnected facilities, including nursing homes and personal residences. Therefore, the presence of only one type of plasmid backbone carrying *vanA* among these patients was striking. Our data are consistent with another recent analysis of VREfm from the United States. Chilambi et al. (34) analyzed gastrointestinal and blood VREfm isolates from pediatric patients at St. Jude’s Children’s Research Hospital over a 10-year period. VanA-type VREfm was isolated from 23 of 24 patients analyzed, and for all 23, Tn*1546* was carried on a *rep17* element. Clearly, these plasmids are significant vectors for vancomycin resistance in E. faecium, as proposed by Freitas et al. (10) in a multilevel analysis of VREfm. In our study, multiple predicted antibiotic resistance genes were detected on these plasmids, in addition to vancomycin resistance genes. The pRUM-like plasmids should be a focus of plasmid surveillance. Resistance genes may be consolidating on this specific backbone, as opposed to the many other plasmids detected in VREfm in this study.

We detected carriage of up to 12 extrachromosomal elements in the VREfm isolates in our study. This is consistent with the comprehensive work of Arredondo-Alonso et al., who conducted a large-scale analysis of the E. faecium plasmidome (35). The authors determined that E. faecium isolates from hospitalized patients possess the largest plasmidome in terms of plasmid number and total base pairs. The striking number of extrachromosomal elements among VREfm isolates warrants further investigation into their maintenance, transmission, and functions.

## MATERIALS AND METHODS

### Bacterial strains analyzed in this study.

The isolates used in this study (Data Set S1A) were cultured from surveillance rectal swabs of high-risk patients on hospital admission (August 2015 to October 2015). Their clinical collection and initial characterization were previously reported (16) and are briefly summarized here. Research involving human subjects was approved by the University of Texas at Dallas and Methodist Hospital (UT-Dallas protocol number MR 14-448 and Methodist Health System protocol number P15MHS.0001A). Rectal swabs from patients admitted with at least one risk factor (hospitalization for ≥2 consecutive nights in the preceding 30 days, transfer from another medical facility, residence in a nursing home or extended/long-term care facility, or the presence of decubitus ulcer or a draining wound) were cultured on Spectra VRE (Remel) agar as a part of routine infection control surveillance. Instead of disposal after clinical procedures were complete, cultures positive for presumptive VRE growth were coded numerically, deidentified, and transferred to the University of Texas at Dallas for further analysis. Isolates were coded by deidentified patient number. If multiple colonies were collected from a Spectra VRE plate, this was indicated by hyphenated numerics. For example, VRE isolates 124-1, 124-2, and 124-3 were collected from the same rectal swab sample from the same patient (deidentified patient number 124). No patients were sampled longitudinally.

In our previous study of these isolates, presumptive VREfm isolates were confirmed by *ddl* typing and for the presence of *vanA* or *vanB* genes by PCR (16, 36). All isolates used in this study, with the exception of 163-1, initially grew on brain heart infusion (BHI) agar supplemented with 256 μg/mL of vancomycin. The vancomycin MIC of isolate 163-1 was previously determined to be 2 μg/mL by broth microdilution (16). The vancomycin MICs of 10 additional isolates were previously determined to be >256 μg/mL by broth microdilution (16). The vancomycin MICs of all other isolates were determined in this study using the same broth microdilution method as previously reported (16).

### Genome sequencing and assembly.

Methods for DNA isolation, MinION sequencing, Illumina sequencing, and hybrid genome assembly are described in Text S1.

### Phylogenetic analyses and MLST.

Genome assemblies were annotated with PROKKA (v 1.12) (37). A sequence alignment was generated for 1,706 core genes by Roary (v 3.12) (22) using PRANK where the paralogs were not split. A phylogenetic tree was built by RAxML (v 8.2.12) (38) with rapid bootstrapping (1,000 inferences) using the GTR+GAMMA model and subsequent ML search on the core gene alignment where the outgroup was specified to be the reference genome E. faecium 1,231,501 (GenBank accession number NZ_ACAY00000000). The phylogenetic tree was visualized with iTOL (v 4) (39). MLST was determined by the E. faecium MLST database (https://pubmlst.org/efaecium/) (7, 40). The novel sequence type ST1703 and novel *gyd* allele number (allele 70) were assigned for the 5 VREfm isolates 111, 121, 137, 154-1, and 158 by request from the database.

### Plasmid detection and typing.

Each contig of <2 Mbp in a closed genome assembly was considered a putative plasmid. *In silico* detection and typing of plasmids were performed with PlasmidFinder (v 2.1) (18). Putative plasmids in closed genome assemblies that were not classifiable by PlasmidFinder were analyzed further (see below).

For comparison with pRUM-like/*rep17* plasmids from Dallas isolates, plasmids were extracted from PLSDB v 2021_06_23_v2 (19, 20) with the following terms: genus = *Enterococcus*, topology = circular, PlasmidFinder = contains *rep17*. Plasmids were visualized with Easyfig (41).

### Definition of pMIX plasmid groups.

Putative plasmid sequences that were unclassifiable by PlasmidFinder were analyzed as follows. Presumptive *rep* genes were identified from plasmid annotations and analyzed by NCBI conserved domain analysis. Confirmed *rep* genes were those that encoded protein domains that matched known Rep protein families with E values of e^−10^ to e^−78^. The Rep sequences were also scanned by HMMER (v 2.41.1) (42) to analyze Pfam domains. Multiple-sequence alignment of the nucleotide and predicted amino acid sequences of *rep* genes was performed with MUSCLE (v 3.8.424) where the “group sequences by similarity” option was selected. A neighbor-joining tree was built based on the sequence alignment using the “Tamura Nei” genetic distance model (43). The tree was visually scrutinized in order to sort the plasmids into 9 groups harboring *rep* genes with at least 95% identity in both nucleotide and predicted amino acid sequence (except for pMI4; see Results). Each unique *rep* group of plasmids was given a name of format pMIX, where the “X” is a numeric number from 1 to 9. A representative sequence from each pMIX *rep* group is in Data Set S1B. Representative sequences were compared to PLSDB v 2021_06_23_v2 (19, 20) using BLASTn with 90% identity and query coverage thresholds.

Eight closed circular DNA elements of various sizes (3 to 72 kb) could not be classified by PlasmidFinder or by the pMIX *rep* typing scheme described above. Analysis of these elements is described in Text S1.

### ANI analysis.

All-versus-all average nucleotide identity (ANI) was calculated using ANIclustermap v 1.1.0 at default parameters with either all E. faecium assemblies or *rep17*/pRUM plasmids as input.

### Transcriptional activity of predicted TA systems.

Transcriptional activity of the toxin-antitoxin (TA) systems, TA*_axe-txe_* and TA*_relE_*, was determined by reverse transcription-quantitative PCR (RT-qPCR) for the VREfm isolates 1 and 5, described in Text S1. The primer sequences used for qPCR are provided in Data Set S1C.

### Resistance gene identification.

Acquired antimicrobial resistance genes were detected with ResFinder 3.1 (44).

### Tn*1546* analysis.

The Tn*1546* nucleotide sequence described for E. faecium BM4147 (GenBank accession number M97297) was used as a reference to identify variations occurring in the Tn*1546* elements identified in this study. The Tn*1546* elements were classified using a previously described nomenclature (13). The presence of insertion sequence elements (IS elements) was indicated with a one-letter code as follows: IS*1216*, B; IS*1251*, C; IS*Efa5*, D; IS*256*, J; IS*Efa16*, K; IS*Efa17*, L; and IS*Efa18*, M. The combination of IS elements within the transposon is described by a two- or three-letter code; e.g., group BC possesses one each of IS*1216* and IS*1251*. Arabic numerals, following the alphabet code, indicate differences due to point mutations or different IS element insertion sites. For the group BC, a previous study (13) described BC1 to BC5, each with specific insertion sites of IS elements. Any novel insertion sites identified in the present study were therefore numbered from BC6 forward. The novel IS*Efa16*, IS*Efa17*, and IS*Efa18* were identified in this study and registered in the ISFinder public database (45).

### Accession number(s).

DNA sequences from this study have been deposited under BioProject PRJNA682584. Individual accession numbers for all sequence files are provided in Data Set S1A.
