# Supplementary figures and images for: Vancomycin Resistance in Enterococcus faecium from the Dallas, Texas, Area Is Conferred Predominantly on pRUM-Like Plasmids
Source: mSphere. 2023 Mar 20;8(2):e00024-23. doi: 10.1128/msphere.00024-23 (PMC10117061; doi:10.1128/msphere.00024-23)

DNA alignment

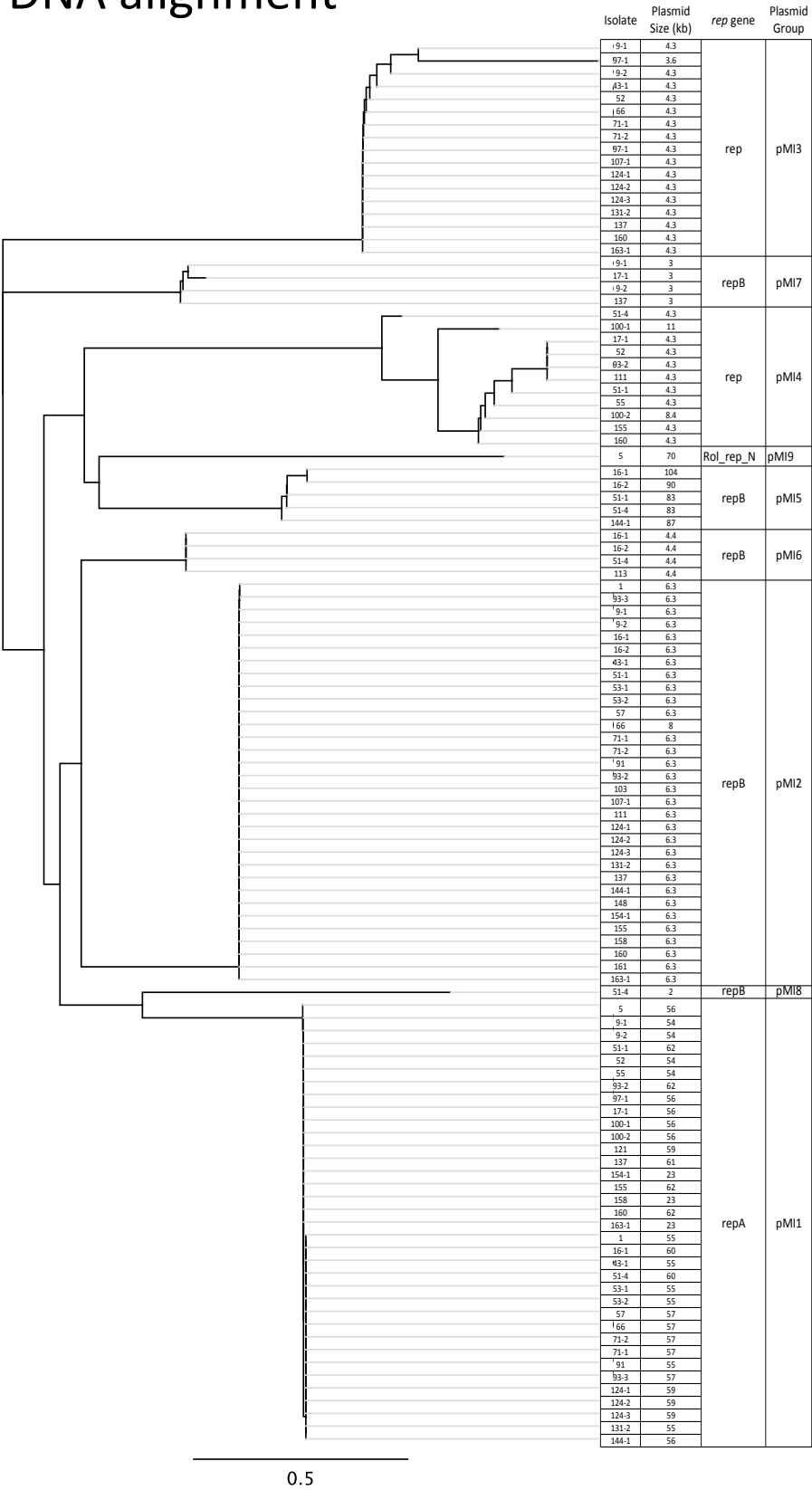

2  
3

Amino acid alignment

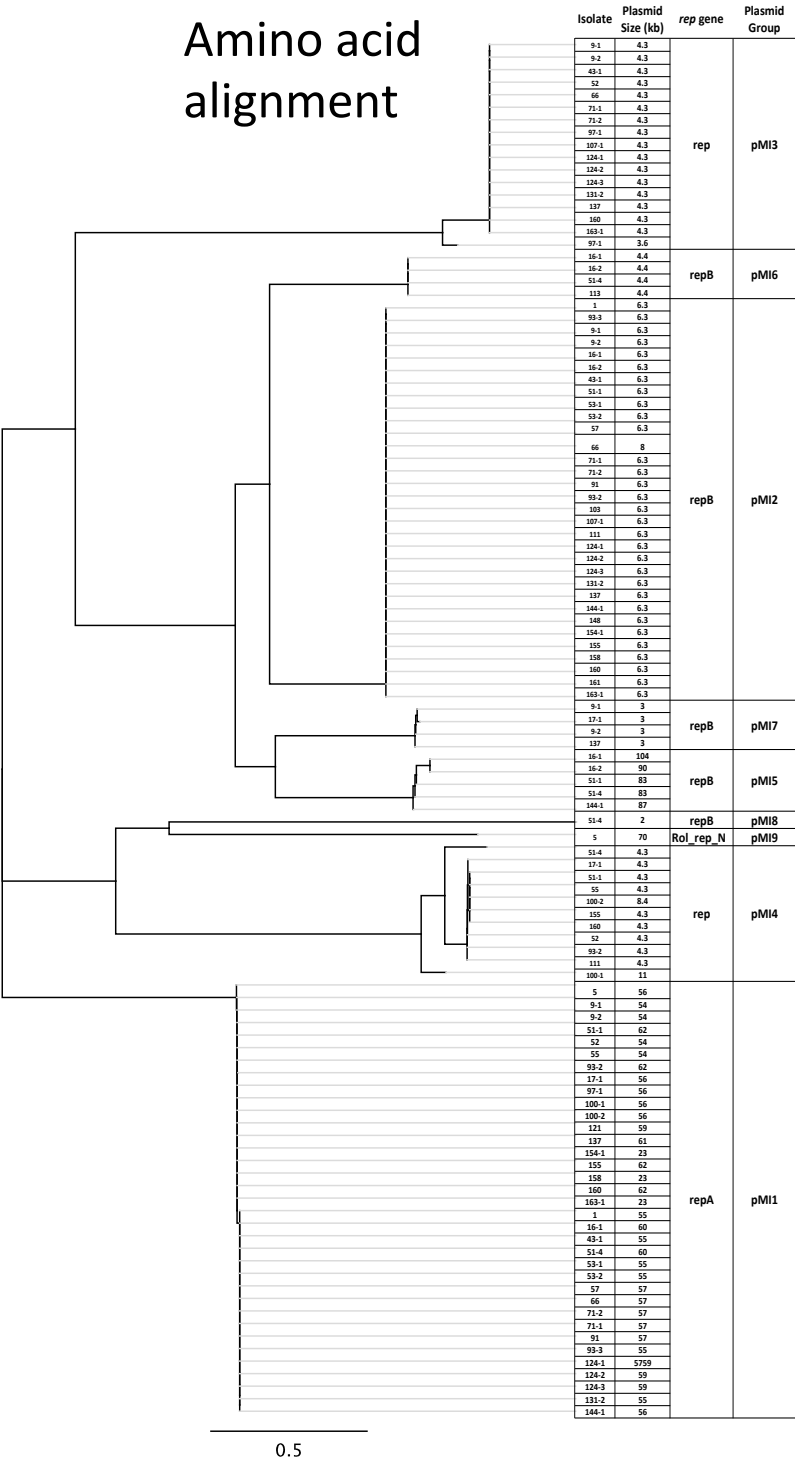

4  
5  
6

Supplement: FIG S1 [file msphere.00024-23-s0002.pdf]

1

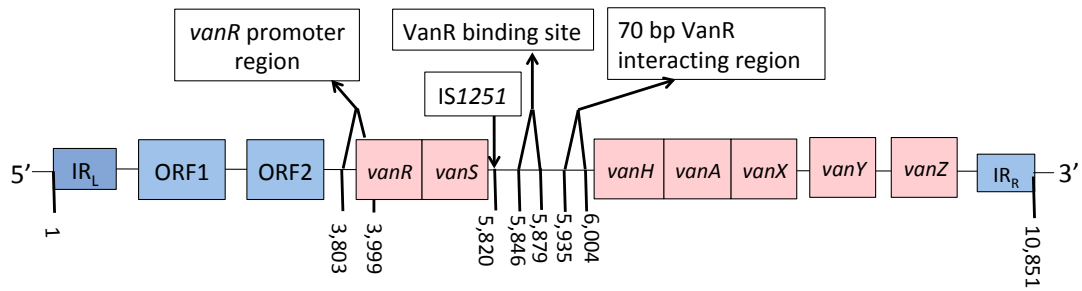

2  
3  
4  
5

Supplement: FIG S2 [file msphere.00024-23-s0003.pdf]

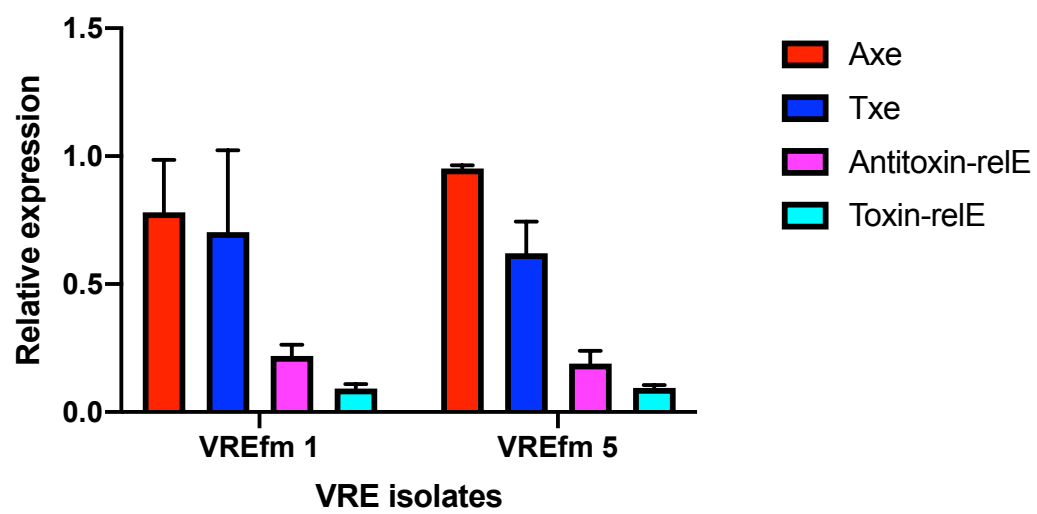

1  
2  
3  
4

Supplement: FIG S3 [file msphere.00024-23-s0004.pdf]

1

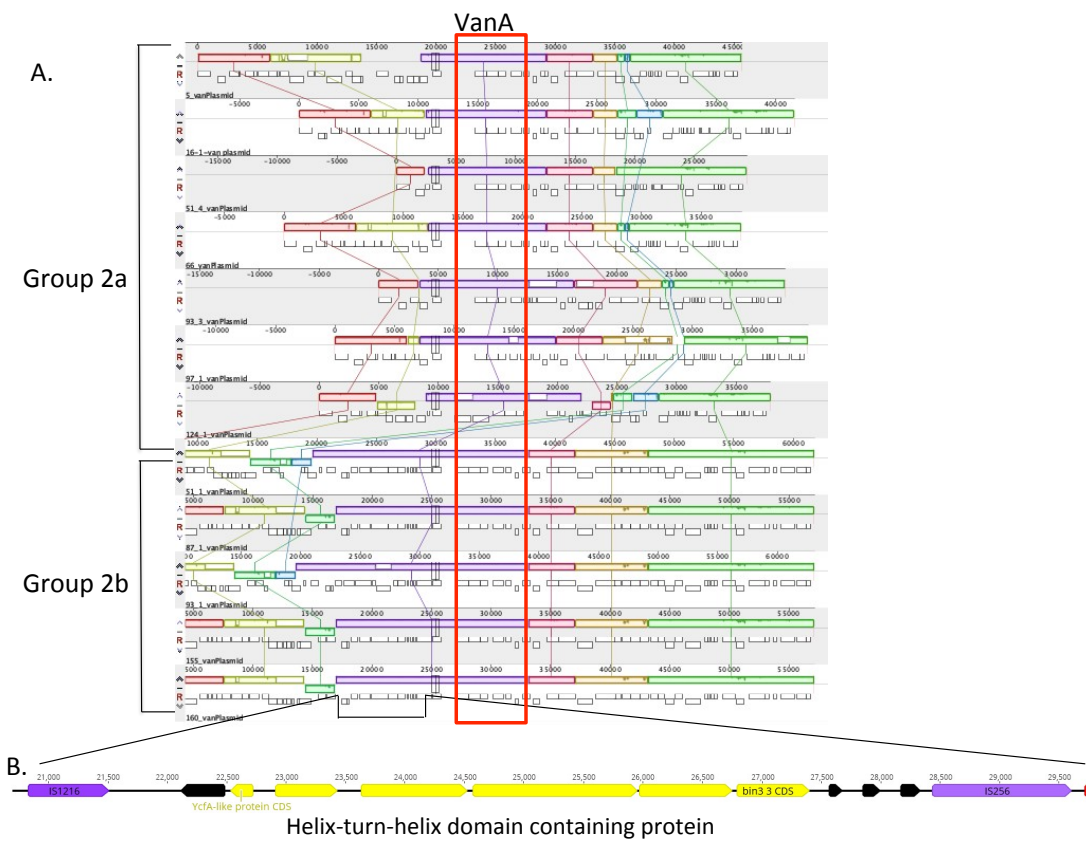

2

3

4

Supplement: FIG S4 [file msphere.00024-23-s0005.pdf]

1

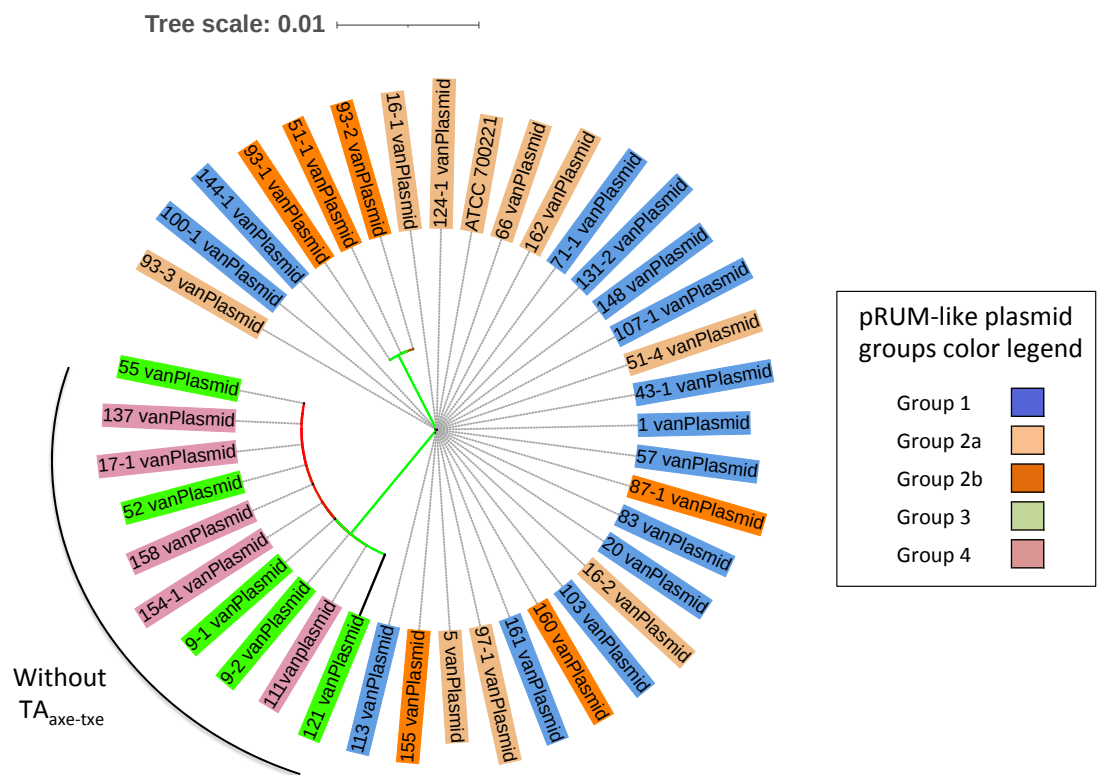

2  
3  
4

Supplement: FIG S5 [file msphere.00024-23-s0006.pdf]

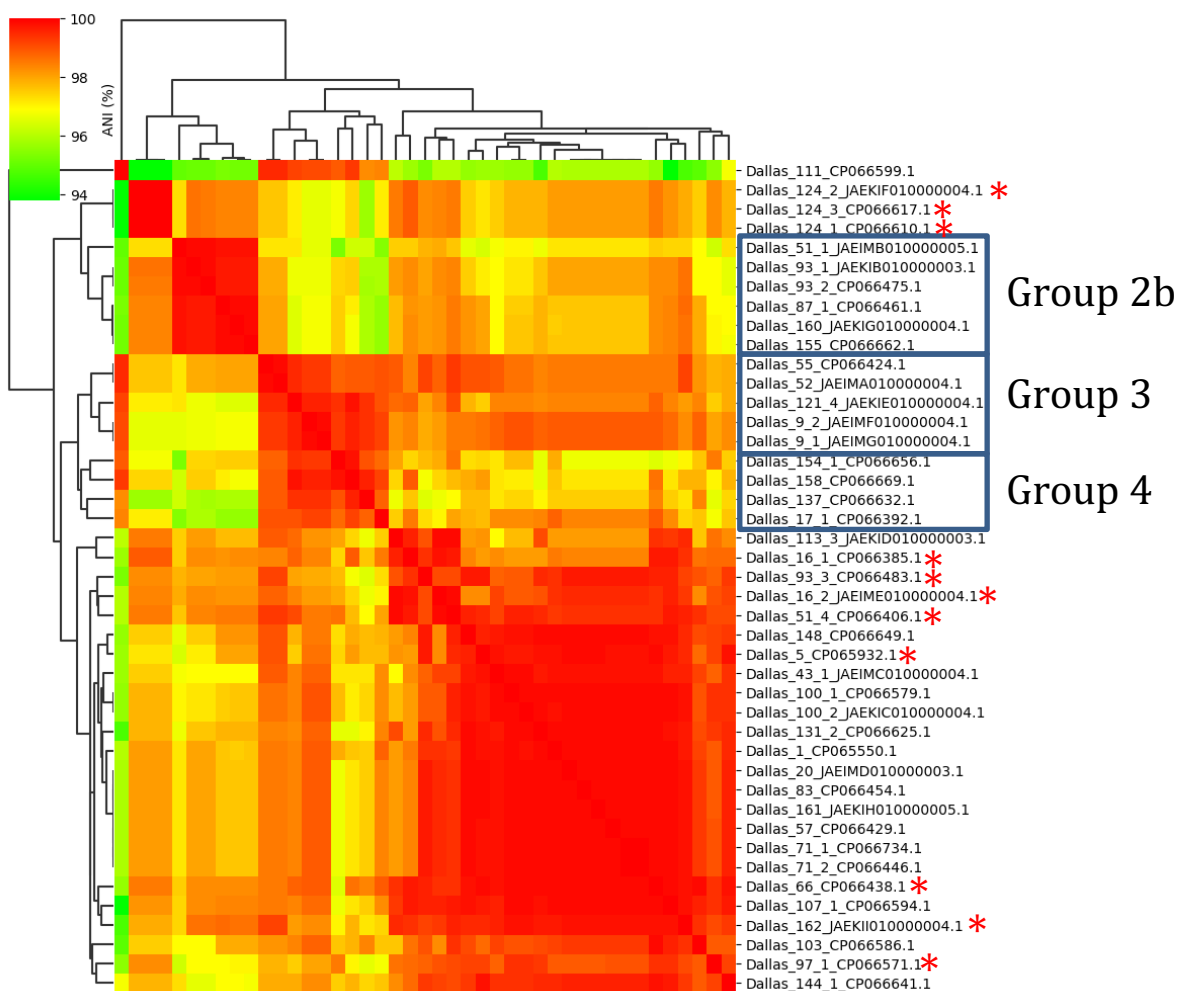

1

2

Supplement: FIG S6 [file msphere.00024-23-s0007.pdf]

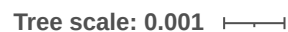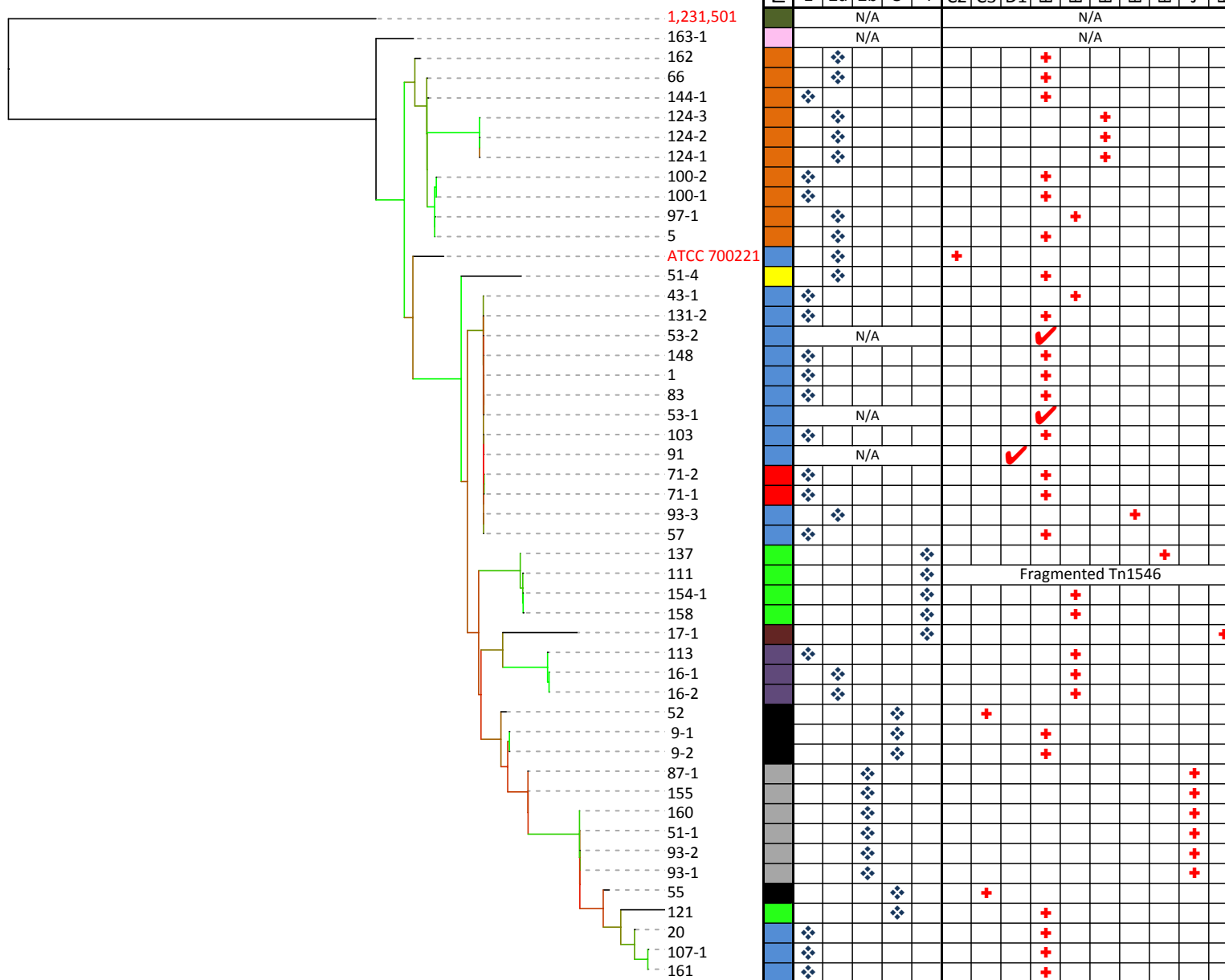

Supplement: FIG S7 [file msphere.00024-23-s0008.pdf]
